# Electromagnetic Reconfiguration Using Stretchable Mechanical Metamaterials

**DOI:** 10.1002/advs.202203376

**Published:** 2023-01-04

**Authors:** Maria Sakovsky, Jan Negele, Joseph Costantine

**Affiliations:** ^1^ Department of Mechanical and Process Engineering ETH Zurich Leonhardstrasse 21 Zurich 8092 Switzerland; ^2^ Department of Electrical and Computer Engineering American University of Beirut Beirut 1107‐2020 Lebanon; ^3^ Present address: Department of Aeronautics and Astronautics Stanford University Stanford CA USA

**Keywords:** aerospace structures, functional materials, stretchable antennas, wearable electronics

## Abstract

Response to environmental thermomechanical inputs in applications that range from wearable electronics to aerospace structures necessitates agile communication systems driven by reconfigurable electromagnetic structures. Antennas in these systems must dynamically preserve acceptable radiation characteristics while enabling on‐demand performance reconfiguration. However, existing reconfiguration mechanisms through stretchable conductors rely on high‐strain behavior in soft substrates, which limits their applicability. Herein, this work demonstrates the use of mechanical metamaterials for stretchable conductors and dielectrics in antennas. Metamaterials allow conductor stretching up to 30% with substrate base material tensile moduli ranging from 26 MPa to 44 GPa. It is shown, through several antenna designs, that mechanical metamaterials enable similar frequency reduction upon stretching as monolithic conductors, while simultaneously providing a miniaturization effect. The conductor patterning, furthermore, provides control over coupling between mechanical stretching and electromagnetic reconfiguration. This approach enables designing reconfigurable antenna functionality through metamaterial geometry in response to arising needs in applications ranging from body‐adapted electronics to space vehicles.

## Graphical abstract

Reconfigurable antennas are realized using stretchable mechanical metamaterials fabricated from soft polymers or stiff fiber reinforced polymer composites. The work demonstrates the control that mechanical metamaterials enable over the coupling between mechanical deformation and electromagnetic functionality. The scalability of the technique targets applications ranging from wearable electronics to satellite communications allowing robust response to environmental thermomechanical stimuli.

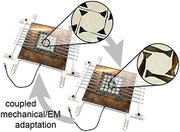

## 1 Introduction

Large shape changes stemming from thermomechanical environmental inputs are inherent to many modern communications systems. As a result, research has focused on the relationship between mechanical deformation and antenna functionality. The flexible conductors constituting sensors and antennas in wearable electronics, for example, have allowed increased user comfort and device durability,^[^ ^1^, ^2^, ^3^ ^]^ while maintaining constant performance in response to stretching due to user motion. Alternatively, systems that react to deformation by adapting their performance have been proposed, thereby creating devices responsive to their environment. For antennas, the conductor geometry is intrinsically tied to its performance, allowing researchers to vary the frequency,^[^ ^4^, ^5^, ^6^, ^7^ ^]^ radiation pattern,^[^ ^8^, ^9^, ^10^, ^11^, ^12^ ^]^ or polarization^[^ ^13^ ^]^ characteristics of the devices and even entirely change the antenna type^[^ ^14^ ^]^ in response to mechanical loading. Frequency‐selective surfaces have employed a similar strategy for on‐the‐fly performance reconfigurability of thin sheets which reject or pass desired electromagnetic frequencies.^[^ ^15^, ^16^, ^17^ ^]^

To enable the required conductor deformation in such applications, a variety of approaches have been proposed.^[^ ^18^ ^]^ Wearable electronics rely predominantly on flexible fabrics coated with thin conductive films^[^ ^1^, ^3^, ^19^ ^]^ or soft polymeric membranes with embedded conductive particles.^[^ ^20^, ^21^, ^22^ ^]^ While achieving large dimensional changes of the conductive surface (as high as 1000%^[^ ^22^ ^]^), this approach sacrifices mechanical properties and cannot be scaled to structural applications. In another technique, electromagnetic metamaterials composed of periodic arrays of radiating elements placed on the rigid facets of foldable origami and kirigami^[^ ^23^, ^24^ ^]^ or substrates with embedded compliant mechanisms^[^ ^25^, ^26^, ^27^ ^]^ enable reconfiguration of communications antennas, filters, and even optical properties. The metamaterials in these examples provide mechanical support for conductors that solely undergo rigid body motion. At a larger scale, antenna reconfiguration for communications and radar applications relies on rigid engineering mechanisms,^[^ ^5^, ^11^, ^12^, ^13^, ^28^ ^]^ smart materials,^[^ ^8^, ^10^ ^]^ or origami techniques.^[^ ^14^, ^15^ ^]^ Once again, a trade‐off between conductor flexibility, mechanical performance, and the physical scale of the system is observed. Consequently, the range of applicability of the various techniques is limited.

To address this gap, we propose a novel approach that relies on mechanical metamaterials as functional substrates for electromagnetic surfaces. Mechanical metamaterials contain cellular geometries that realize homogenized properties distinct from those of their base materials and can be engineered to achieve large deformations.^[^ ^29^, ^30^ ^]^ The geometry frequently relies on the bending of slender elements to achieve extreme global stretching up to 400% elastically, significantly exceeding the elastic limits of their base material.^[^ ^31^, ^32^ ^]^ This design principle is unique in that it leads to scale‐ and material‐independence. Existing implementations utilize geometric features spanning from the nanometer scale^[^ ^33^, ^34^, ^35^ ^]^ to the centimeter scale^[^ ^36^, ^37^, ^38^ ^]^ and base materials with moduli spanning 7 orders of magnitude (from 500 kPa to 200 GPa).^[^ ^33^, ^36^, ^39^, ^40^ ^]^ As such, the approach is promising for conductor reconfiguration beyond rigid body motion and is scalable compared to flexible conductors integrated into elastomeric substrates.

**Figure 1:**
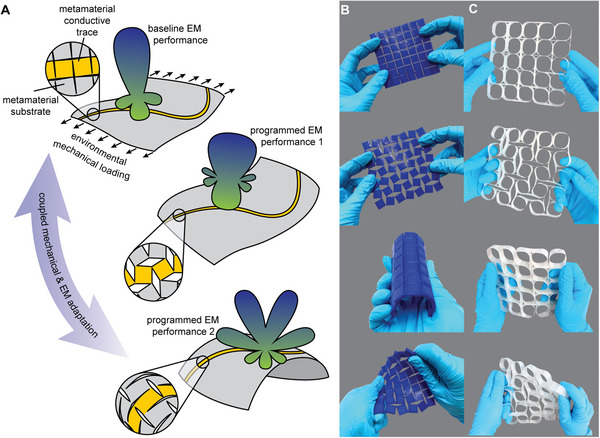
Stretchable metamaterial antenna concept and material independence. A) A schematic illustrating use of metamaterial substrates selectively coated with conductive traces as reconfigurable antennas. We leverage environmental mechanical loading that leads to substrate and conductor deformation to achieve adaptive antenna performance. B) A 3D‐printed metamaterial substrate from thermoplastic polyurethane. From top to bottom: undeformed, in‐plane stretching, bending, twisting. C) A shell‐based metamaterial substrate manufactured from glass fiber reinforced epoxy. From top to bottom: undeformed, in‐plane stretching, bending, twisting.

We demonstrate the potentials of the proposed approach for reconfigurable electromagnetic surfaces using sheet‐like dielectric substrates patterned into a metamaterial geometry allowing high in‐plane deformation. The substrates are selectively coated with a thin conductive layer on one side. Stretching of the metamaterial results in a change of geometry of the conductor. Employing this for an antenna radiating surface allows adaptation of antenna performance in response to an external mechanical loading (**Figure** **1**A). The precise mechanical metamaterial topology, geometric parameters, and base material offer a rich design space for controlling the coupling between mechanical deformation and antenna performance.

This study leverages the scalability inherent to mechanical metamaterials for application to frequency reconfigurable antennas demonstrating that we can balance conductor flexibility, structural properties, and control physical scale. In this scope, we address the effects of the metamaterial patterns introduced into the dielectric substrates and conductors on the antenna performance, show the applicability of the methodology to various antenna operating principles, and present a design approach to tailor the frequency change for a given applied strain. We demonstrate unique tuning of the coupling between mechanical stretching and change in electromagnetic performance as the metamaterial patterning of both the conductive and dielectric elements can be used for control. In combination with the material‐independence of the approach, this can be leveraged for a broad range of applications from soft wearable electronics where electromagnetic reconfiguration is driven by user motion to lightweight, load‐carrying satellite antennas that respond to the changing thermal environment on orbit.

## 2 Design Space of Mechanical Metamaterials

A fixed rotating square metamaterial topology, consisting of a grid of square segments interconnected via slender hinges, was selected for this study (Figure 1B). In the idealized case of rigid segments, the pattern yields auxetic behavior with a Poisson's ratio of *ν* = − 1.^[^ ^41^ ^]^ This auxetic behavior is required for the substrate and conductive trace to maintain geometric similarity upon uniaxial stretching. This is an appropriate choice for frequency reconfiguration as the operating frequency in antennas is inversely proportional to the dimensions of a conductive surface. However, the presented approach is broadly applicable to a variety of mechanical metamaterial topologies, where the Poisson's ratio of the substrate dictates the electromagnetic performance metric undergoing reconfiguration (Section S1, Supporting Information).

The in‐plane compliant mode of the metamaterial is enabled through the bending of the slender hinges connecting the segments. Thereby, large global deformations are possible while keeping material strains within their elastic limits. This enables material‐independence when designing stretchable antennas. For example, the substrate can be 3D printed as a thin sheet of soft polymer (see the Experimental Section), with both in‐plane and out‐of‐plane compliance (Figure 1B). The large surface area of the segments provides a surface for application of a thin conductive film. This substrate conforms to a variety of 3D surfaces, such as those found in wearable electronics.

Alternatively, the substrate can be fabricated from thin fiber reinforced polymer (FRP) composite shells (Figure 1C; Experimental Section). These base materials are axially stiff but can undergo large deformations in bending due to the small material thickness.^[^ ^42^ ^]^ The topology is identical to that of the polymer design with the exception that the segments are hollow due to the shell structure. Non‐uniform base material thickness distribution yields the desired *ν* = − 1 behavior for a large range of applied strains (Section S2, Supporting Information; Experimental Section). The resulting substrate has in‐plane compliance but high out‐of‐plane bending stiffness due to the large substrate thickness and high axial stiffness of the FRP. In fact, previous studies by the authors of a similar metamaterial topology have shown in‐plane expansion up to 60% before material failure.^[^ ^39^ ^]^ This makes it ideally suited for realizing ultra‐lightweight, yet load‐carrying, antenna structures, for example, those found in aerospace applications.^[^ ^43^, ^44^ ^]^ A thin conductive layer can be bonded to the top surface of the substrate to realize antenna functionality (see the Experimental Section).

Here, we leverage the material independence to realize metamaterial substrates from base materials with very different tensile moduli, *E*: a soft thermoplastic polyurethane (TPU) (*E* = 26 MPa) and a glass fiber reinforced epoxy polymer (*E* = 44 GPa in fiber direction with 60% fiber volume content).

## 3 Metamaterial Substrates for Frequency Reconfigurable Wire Antennas

We first consider the application of the mechanical metamaterial concept to frequency reconfigurable wire antennas. In this simple case, the metamaterial dielectric plays a role only as a structural support.

**Figure 2:**
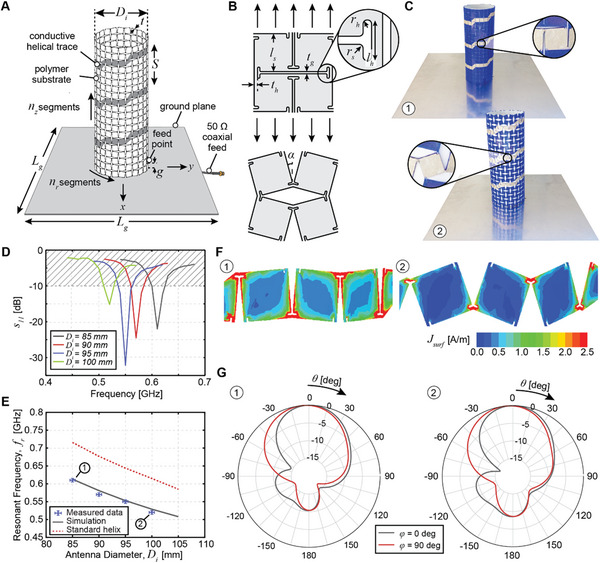
Frequency reconfigurable helical wire antenna with TPU metamaterial substrate. A) Schematic of metamaterial helical antenna. The metamaterial cylinder is composed of *n*_z_ × *n*_r_ segments across its height and circumference, respectively. A helical trace with *n*_turns_ turns is coated onto the cylinder using conductive ink. B) Rotating square metamaterial topology in the undeformed and stretched configurations. The geometry is parameterized via the segment side length, *l*_s_, corner radius, *r*_s_, the connecting hinge length, *l*_h_, thickness, *t*_h_, and radius, *r*_h_, and the gap between segments, *t*_g_. Uniaxial stretching results in segment rotation by angle *α*. C) The prototype is pictured in its undeformed 1) and stretched 2) configurations. For this antenna: *l*_s_= 10.2 mm, *t*_h_= 0.6 mm, *t*_g_= 0.2 mm, *r*_h_= 0.2 mm, *l*_h_= 2.0 mm, *r*_s_= 0 mm, *t* = 1.2 mm, *L*_g_= 400 mm, *n*_turns_= 3.2, and *n*_r_ × *n*_z_ = 24 × 20. D) Measured reflection coefficient magnitude for several helix configurations. E) Demonstration of operating frequency reconfiguration upon stretching. Error bars represent experimental uncertainty of measurement. F) Simulated surface current distribution with 0° phase in undeformed 1) and deformed 2) configurations. G) Radiation pattern elevation cuts in undeformed 1) and deformed 2) configurations provided at the respective resonant frequency.

A helical wire antenna is realized from a 3D‐printed TPU metamaterial cylinder of inner diameter, *D_i_*, and thickness, *t*, used as a support for a conductive helical trace positioned on top of a square ground plane of side length, *L*_g_ (**Figure** **2**A). The rotating square metamaterial geometry is parameterized as in Figure 2B. The helical trace is continuous and follows the metamaterial pattern of the supporting cylinder. The antenna is fed with a 50 Ω coaxial cable attached at a distance *g* above the ground plane. The helix is designed to operate in an axial mode and the helix spacing, *S*, is selected such that *S* = *π*(*D*_i_ + 2*t*)/4 to minimize losses.^[^ ^45^ ^]^

The helix is pictured in its undeformed and stretched configurations in Figure 2C, with the patterning of the conductive trace highlighted. By circumferentially stretching the cylinder, a reduction of the operating frequency of the antenna is obtained, with excellent agreement between experimental measurements using a vector network analyzer and numerical simulations using ANSYS Electronics Desktop^[^ ^46^ ^]^ (Figure 2E; Experimental Section). Due to a Poisson's ratio of *ν* = − 1, the helix maintains geometric similarity upon expansion and the antenna remains well matched to the 50Ω feed line upon stretching, with a reflection coefficient magnitude, *s*_11_ < −10 dB, for a roughly 4%–5% fractional bandwidth in each configuration (Figure 2D).

We define a metric, *η*_*ε*_, for characterizing the average operating frequency, *f_r_*, change for a given change in characteristic length of the antenna, *L*_c_,

(1) $$\eta_{\varepsilon }=\frac{\Delta f_{r}}{\varepsilon }\;=\;\frac{f_{r}^{*}-\;f_{r}^{0}}{f_{r}^{0}}\frac{L_{c}^{0}}{L_{c}^{*}-L_{c}^{0}}$$

where the superscripts ^0^ and * indicate values at the undeformed and deformed configurations, respectively, and *ε* is the applied strain as measured by the characteristic length change.

For the helix, the characteristic length is taken to be the outer diameter of the cylinder, *L*_c_ = *D*_i_ + 2*t*.^[^ ^45^ ^]^ We find that for the metamaterial helix antenna *η_ε=_*_20%_ = − 0.76 (Figure 2E, gray), compared to *η_ε=_*_20%_ = − 0.78 for a standard monolithic helix with the same diameter, spacing, and height (Figure 2E, red). The close correspondence indicates that despite the patterning of the conductor, the use of metamaterials for changing the characteristic length of this wire antenna is as efficient as the use of a monolithic stretchable conductor. This is a very important feature, allowing us to leverage the various advantages of metamaterial antennas while achieving acceptable antenna performance.

The conductor's patterning is furthermore advantageous due to a miniaturization effect seen for the metamaterial antenna relative to a standard helix of the same size (Figure 2E). This can be explained by the current path topology as it flows around the segment edges (Figure 2F). Hence, the antenna with patterning exhibits a lower frequency operation for the same physical size. The effect is analogous to the miniaturization principle that can be observed in fractal antennas.^[^ ^45^, ^47^ ^]^

Lastly, the axial mode operation of the helix is maintained throughout stretching (Figure 2G). We find a peak realized gain of 6.2 dB in the undeformed configuration with a slight reduction to 5.7 dB upon stretching to *D*_i_ = 100 mm. The gains can be improved through tailoring of mechanical metamaterial topology (Section S1, Supporting Information).

## 4 Metamaterials as Dual‐Function Structural and Electromagnetic Elements

**Figure 3:**
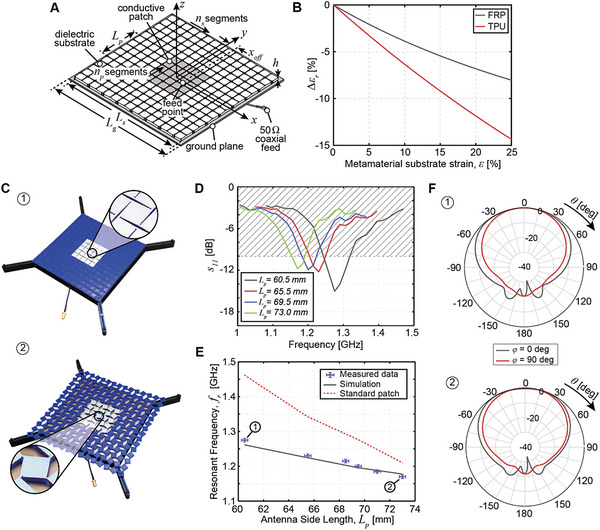
Frequency reconfigurable patch antenna with a TPU substrate. A) Schematic of mechanical metamaterial square patch antenna. The geometry is parameterized by the side lengths of the ground plane, *L*_g_, substrate, *L*_s_, and conductive patch, *L*_p_, and the substrate height, *h*. The metamaterial substrate consists of *n*_s_ × *n*_s_ segments and the conductive patch spans *n*_p_ × *n*_p_ segments. B) Dependence of substrate dielectric constant on metamaterial stretching. C) Photographs of the TPU patch in undeformed 1) and deformed 2) configurations. The prototype geometry is as follows: *l*_s_ = 11.4 mm, *t*_h_ = 0.6 mm, *t*_g_ = 0.2 mm, *l*_h_ = 2.0 mm, *r*_h_ = 0.2 mm, *r*_s_ = 0 mm, *h* = 3.1 mm, *L*_g_ = 180 mm, *n*_s_ = 5, *n*_p_ = 15, and *x*_off_ = 12.0 mm. D) Measured reflection coefficient magnitude for several patch configurations. E) Demonstration of operating frequency reconfiguration upon stretching. Error bars represent the experimental uncertainty of measurement. F) Radiation pattern elevation cuts in undeformed 1) and deformed 2) configurations provided at the respective resonant frequency.

Next, we consider more complex antenna types, where the metamaterial dielectric plays both a structural and an electromagnetic function. Specifically, we demonstrate the versatility of the mechanical metamaterial approach through its application to a frequency reconfigurable square patch antenna (**Figure** **3**A). The patch consists of a metamaterial dielectric, selectively coated with a conductive layer to realize a square patch and set on top of a ground plane. The antenna is fed at (*x*_off_, 0) to achieve impedance matching to a 50 Ω coaxial cable. Here, the dielectric substrate acts not only as a structural support but also has an influence on the electromagnetic performance through its dielectric material properties.

For a rectangular patch, the resonance frequency can be estimated as a function of the patch length, *L*_p_, the substrate dielectric constant, *ε*_r_, and a fringe factor, *q*, which accounts for the fringing of the electric field at the patch edges and is proportional to the dielectric height, *h*,^[^ ^45^ ^]^

(2) $$f_{r}=\;q\frac{1}{2L_{p}\sqrt{\varepsilon_{r}}\sqrt{\mu_{0}\varepsilon_{0}}}$$

where *µ*_0_ and *ε*_0_ are constants representing the vacuum permeability and permittivity, respectively.

We discuss the effects of stretching the mechanical metamaterial patch in the framework of Equation 2. First, the increase in *L*_p_ due to the stretching of the substrate is expected to reduce the operating frequency. Second, as the antenna is stretched, air gaps are introduced into the dielectric substrate. Using an analytical model of parallel capacitances to estimate the effect this process has on the dielectric properties of the substrate (Section S3, Supporting Information), we find a drop in *ε*_r_ as the antenna is stretched and contains increasingly more air by volume. Comparing the two substrates in this study (Figure 1B,C), this effect is minimized for the FRP metamaterial substrate as its shell‐based design is predominantly air in all configurations (Figure 3B). The particular FRP substrate in this study is 89%–93% air by volume when stretched from *ε* = 0% to *ε* = 25%. As per Equation 2, reduction of the dielectric constant is expected to increase the operating frequency upon stretching, competing with the effects of increased patch length. Lastly, changes in the substrate's material distribution upon stretching also impact the fringing of the electric fields. This is not considered in the analytical model but is accounted for by the high fidelity finite element simulations of patch performance. It is expected that balancing of these effects gives precise control over the relation between mechanical deformation and operating frequency.

**Figure 4:**
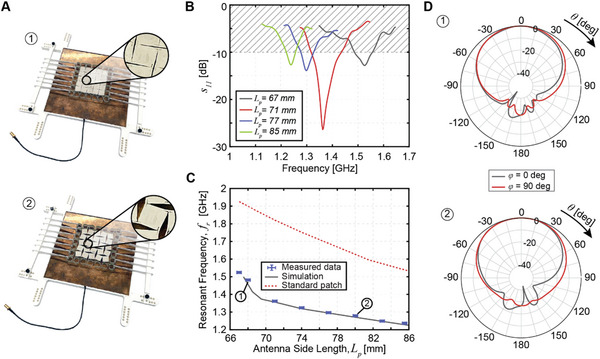
Frequency reconfigurable patch antenna with an FRP substrate. A) Photographs of the FRP patch in two deformed configurations (more details available in Figure S4 in the Supporting Information). The prototype geometry is as follows: *l*_s_ = 13.4 mm, *t*_h_ = 0.15 mm, *t*_g_ = 0 mm, *l*_h_ = 8.6 mm, *r*_h_ = 0 mm, *r*_s_ = 6.1 mm. The conductive patch has identical geometry with the exception that *r*_s_ = 0.5 mm. The antenna has geometry: *h* = 8.0 mm, *L*_g_ = 180 mm, *n*_s_ = 5, *n*_p_ = 7, and *x*_off_ = 19.0 mm. B) Measured reflection coefficient magnitude for several patch configurations. C) Demonstration of operating frequency reconfiguration upon stretching. Error bars represent experimental uncertainty of measurement. D) Radiation pattern elevation cuts in two deformed configurations, 1) and 2), provided at the respective resonant frequency.

Patch antennas are realized using both the TPU (Figure 3C) and the FRP (**Figure** **4**A) metamaterial substrates. The competing effects of the increase in *L*_p_ and reduction of *ε*_r_ upon stretching are evident in the different responses of the two antennas (Figures 3E and 4C). In both cases, the stretching of the conductor has a higher impact than a reduction of the substrate dielectric constant, leading to an overall reduction of frequency upon stretching. Impedance matching of the prototypes is maintained upon stretching with an input impedance of 50Ω and a measured reflection coefficient magnitude *s*_11_ < −10 dB for a fractional bandwidth of 3%–6% in each antenna configuration, for both TPU (Figure 3D) and FRP (Figure 4B) designs.

Evaluating the average frequency change due to a 20% stretch for the TPU patch antenna yields *η*_*ε*=20%_ = − 0.38 (Figure 3E, gray) compared to *η*_*ε* =20%_ = − 0.75 for a standard patch reference of the same dimensions with a monolithic TPU dielectric and conductive layers (Figure 3E, red). In contrast, a much larger frequency change is observed for the FRP patch with *η*_*ε* =20%_ = − 0.81 (Figure 4C, gray). This is comparable to the frequency change of a standard patch antenna of the same dimensions with a full air dielectric of *η*_*ε* =20%_ = − 0.86 (Figure 4C, red). This indicates that metamaterial stretchable patch antennas can be designed as efficiently as monolithic ones for the purpose of antenna frequency reconfiguration through appropriate design of the mechanical metamaterial dielectric. Furthermore, cyclic stretching of the FRP patch antennas does not impact the observed frequency change (Figure S7, Supporting Information).

Similar to the case of the helical antenna, a miniaturization effect is observed in comparison with the standard patch reference antennas (Figures 3E and 4C). Lastly, we note that the radiation patterns of the metamaterial patches are similar to the radiation patterns of the standard patch and are largely unaffected by stretching (Figures 3F and 4D). Experimental measurements of the radiation patterns verify simulations (Figure S5, Supporting Information). The TPU patch achieves a peak realized gain of 1.4 dB in the unstretched configuration and 1.5 dB when stretched to *L*_p_ = 73 mm (*ε* ≈ 20%). The FRP patch achieves much higher values of 8.3 dB in the undeformed configuration and 7.4 dB when stretched to *L*_p_ = 80 mm (*ε* ≈ 20%) due to the large substrate height without a mass penalty. The dielectric substrate and conductive layers of the FRP patch in Figure 4A have an average density of only 0.15 g cm^−3^ compared to 1.85 g cm^−3^ for a printed circuit board typically used for patch antennas.

We show that careful design of the metamaterial dielectric substrate to minimize dielectric property change upon stretching can maximize frequency reduction. While this is a crucial effect to account for during the design process, it is difficult to use this in practice to control of frequency change in response to a given mechanical stretching. In particular, material distribution in the dielectric also creates changes in the fringing of the electric field between the conductive patch and ground plane. As *q* = *f*(*ε*_r_) for mechanical metamaterial substrates, the relation between *f*_r_ and *ε*_r_ is no longer monotonic as in Equation 2. Instead, the metamaterial pattern of the conductive patch itself provides an effective parameter for designing a desired frequency reconfiguration.

## 5 Tailoring Frequency Adaptation through Mechanical Metamaterial Geometry

In addition to providing flexibility on the antenna topology and type, the metamaterial concept also offers significant control of antenna performance through modification of geometric parameters. In particular, we demonstrate the ability to leverage the geometric parameters of the metamaterial conductors in Figure 2B to cater to a desired change in the antenna's operating frequency in response to mechanical stretching.

We find that the most critical geometric parameters controlling the frequency change metric, *η*_*ε*_, for the patch antenna are the degree of segmentation, $n\;=L_{p}^{0}\;/l_{s}$, and the normalized segment radius, *ρ* = *r*_s_ /*l*_s_. The achieved frequency reconfiguration is relatively insensitive to other changes in geometry (Section S6, Supporting Information).

**Figure 5:**
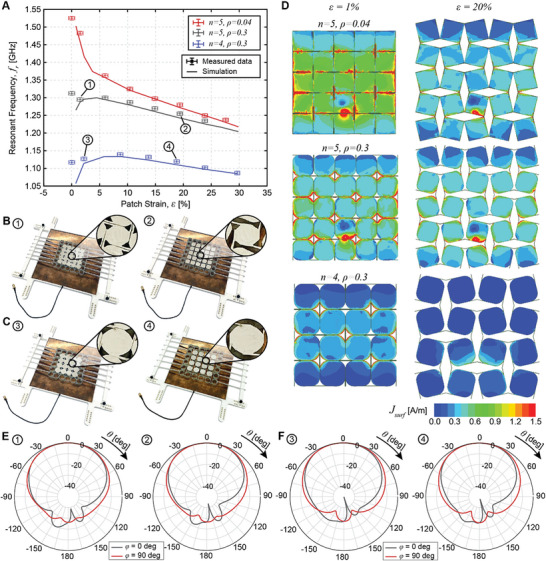
Influence of conductor metamaterial geometry on frequency reconfiguration. A) Operating frequency as a function of strain, *ε*, for three patch antenna prototypes with an FRP dielectric substrate. Error bars represent the experimental uncertainty of measurements. B) Photographs of the prototype with *n* = 5, *ρ* = 0.3 in two stretched configurations, 1) and 2). C) Photographs of the prototype with *n* = 4, *ρ* = 0.3 in two stretched configurations, 3) and 4). D) Comparison of the simulated surface current densities of the patch prototypes in the undeformed (*ε* = 1%) and deformed (*ε* = 20%) configurations. E) Radiation pattern elevation cuts in two deformed configurations, 1) and 2), for the prototype with *n* = 5, *ρ* = 0.3 provided at the respective resonant frequency. F) Radiation pattern elevation cuts in two deformed configurations, 3) and 4), for the prototype with *n* = 4, *ρ* = 0.3 provided at the respective resonant frequency.

Experimental demonstration of the effects of these parameters is provided in **Figure** **5**, which shows the frequency reconfiguration of three antenna prototypes with varying *n* and *ρ* for the conductive patch layer. Note that the prototype with *n* = 5, *ρ* = 0.04 corresponds to the FRP patch antenna in Figure 4. The two prototypes with *n* = 5 have identical dielectric geometries. The dielectric substrates for the two prototypes with *ρ* = 0.3 are of identical geometry to their respective conductors.

An increase of the segment radius, *ρ*, results in a significant reduction of the resonant frequency of the antenna for substrate strains below 10% (Figure 5A, red and gray). It is insightful to compare the patch surface current densities to understand this behavior (Figure 5D). The paths of the surface current densities follow the physical connections between the different segments via the hinges. In addition, it is important to note that portions of these surface current densities are induced current densities from neighboring segments. For low *ρ*, the gaps in the conductor are small and the surface currents at *ε* = 1% resemble that of a standard patch, with a surface current of the dominant operating mode across the patch length. Larger *ρ* values lead to gaps in the conductor, even in the unstretched configuration, thereby reducing the induced current portion of the surface current densities at low strains. In this case, the current path length is increased as the current tends to run across the segment perimeters, lowering the operating frequency. At high substrate stretching, the presiding effect is due to the gaps introduced during metamaterial stretching and the behavior is similar for both prototypes.

A reduction in the degree of segmentation, *n*, results in a change of both the absolute operating frequency and the variation of resonant frequency with substrate stretching (Figure 5A, gray and blue). Lower segmentation results in a further drop in induced currents for low substrate stretching (Figure 5D) and causes the operating frequency to increase before decreasing again as the substrate is stretched.

The change of operating frequency for *ε* > 20% is largely unaffected by geometry, with only a small variation of the slope of the curves in Figure 5A. Only the absolute operating frequency is affected. For *ε* < 20%, control over the frequency change metric, *η*_*ε*_, can be achieved via conductor metamaterial geometry.

The radiation patterns of the FRP patches remain unaffected by the degree of segmentation and segment radius (Figure 5E,F). However, the metamaterial geometry affects the realized patch gains. At low strains, a higher conductor coverage area (higher *n* and lower *ρ*) leads to improved performance with peak realized gains of 8.3, 6.4, and 5.1 dB for the prototypes with *n* = 5, *ρ* = 0.04, *n* = 5, *ρ* = 0.3, and *n* = 4, *ρ* = 0.3, respectively. At high strains, the gain is unaffected by the conductor geometry (7.3–7.4 dB for all prototypes when stretched to *ε* = 20%).

**Figure 6:**
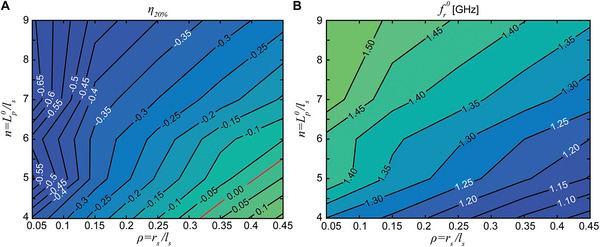
Parametric study demonstrating design for a desired frequency reconfiguration. A) Simulated frequency change metric, *η*_*ε* =20%_, as a function of the patch segmentation, *n*, and the normalized segment radius, *ρ*. B) Simulated operating frequency of the patch in its undeformed configuration as a function of the patch segmentation, *n*, and the normalized segment radius, *ρ*.

To derive a clear design space, unaffected by small manufacturing imperfections, the frequency change metric is extracted for a range of geometries with differing *n* and *ρ* (**Figure** **6**A). For a given *n*, the FRP substrate is fixed and only the metamaterial conductor geometry is varied. The volume fraction of FRP material in the substrate is kept constant for all designs by adjusting the FRP shell thickness as a function of *n*. This aims for a constant change in substrate dielectric constant upon stretching for all designs and allows for evaluation of only the effects of conductor geometry.

The strongest influence on frequency reconfiguration is determined to be from the normalized segment radius, *ρ*. An increase in radius leads to a lower frequency drop. In fact, for high enough values, the frequency can be kept constant upon stretching (red line in Figure 6A) and beyond this, the frequency drop is reversed.

For *ρ* < 0.15, the segmentation has little influence on performance as induced currents remain high at low stretching and the surface current distribution closely resembles that of a solid patch regardless of the segmentation (as in Figure 5D). In this range, low segmentation can be used to reduce fabrication complexity without affecting electromagnetic performance. At higher values of *ρ*, the segmentation begins to play an important role, with an increase of operating frequency due to stretching only possible for low degrees of segmentation.

The absolute operating frequency for the undeformed antenna is inversely proportional to the frequency change metric (Figure 6B). To obtain the desired frequency change in response to a given external loading and a set initial operating frequency, both the normalized conductor geometry and the patch size, $L_{p}^{0}$, can be appropriately selected.

## 6 Discussion and Conclusions

A predominant use case for mechanically reconfigurable antennas over electrical reconfiguration is that they can adapt their electromagnetic performance in response to stimuli in their environment. The mechanical metamaterial approach allows the design of adaptive antennas using a broad range of dielectric substrate materials. Soft dielectric materials (like the TPU design) are ideal for wearable electronics with metamaterial patterning miniaturizing such devices for increased user comfort. By tuning the metamaterial geometry, we can realize devices that maintain their operating frequency and radiation pattern characteristics as the user moves (as is demonstrated by designs with geometric parameters falling on the red line in Figure 6A). Other embodiments can include devices that link user motion to an operating frequency change for the monitoring of physiological data. Tailoring geometric parameters as in Figure 6 can yield sensors with tunable sensitivity for a particular measurement or individual.

Alternatively, our methodology can target structural antenna applications with stringent requirements including low mass, high stiffness, and high antenna gains. The scale of our designs is easily applicable to satellite antenna structures that range from the centimeter scale to the meter scale.^[^ ^43^, ^44^ ^]^ In this case, the frequency reconfiguration can be leveraged for cognitive radio applications^[^ ^48^ ^]^ or to obtain feedback on the shape of a large flexible space structure.^[^ ^49^ ^]^

In this work, we demonstrated the potential of mechanical metamaterials to act as versatile stretchable conductors and dielectrics in physically reconfigurable antenna systems. Since the large achievable stretching is enabled by the metamaterial geometry and does not require high strain materials, the concept has been applied to materials with very different moduli ranging from relatively soft TPU to a stiff glass fiber composite. For the first time, we demonstrate an approach for mechanical antenna reconfiguration that simultaneously allows for conductor flexibility, improved mechanical performance, and a broad range of physical scales.

Mechanical metamaterial conductors can achieve similar frequency reduction in antenna systems upon stretching as monolithic stretchable conductors, such as more established elastomeric conductors. Functionality was shown in a simple helical wire antenna but also a more complex microstrip patch antenna, where the dielectric substrate additionally plays a functional role in the electromagnetic response. Unlike monolithic stretchable conductors, the mechanical metamaterial patterning controls the coupling between mechanical stretching and antenna frequency reconfiguration. The versatility in material choice and topology as well as the tuning ability of the resonance frequency change offered by mechanical metamaterials allows the design of antenna structures that respond to a broad range of thermomechanical stimuli in their environment.

## 7 Experimental Section

### Materials

The polymer antenna substrates were 3D printed from a TPU filament (Ultimaker TPU 95A). The material has an elongation at yield of 55%, allowing high elastic deformations, a density of 1.22 g cm^−3^, and a dielectric constant of 4.12 measured at 1 MHz.^[^ ^50^ ^]^ Conductive traces were realized using a water‐based silver particle solution with a resistivity of 7.5 × 10^−5^ Ω cm (842WB from MG Chemicals).^[^ ^51^ ^]^

The FRP patch antennas were fabricated from thin preimpregnated sheets of E‐glass unidirectional fibers (PPG 2026 600 tex) with an areal weight of 25 g m^−2^ and an epoxy matrix (NTPT ThinPreg402). All prototypes had a 16‐ply [0°_2_/90°_2_/0°_4_]_s_ cross‐ply laminate comprising the rounded segments and a thinner 6‐ply [0°_2_/90°]_s_ laminate for the hinges joining them, where the 90° direction indicates fibers perpendicular to the plane of the substrate and 0° is a local material direction indicating fibers lying in the plane of the substrate and tangent to the shell surface. The stiff composite layup used for the segments relative to the soft layup used for the hinges ensured that the substrate has a Poisson's ratio of −1 (Section S2, Supporting Information).^[^ ^39^ ^]^ The fiber volume content was measured at 0.57 using optical microscopy (Keyence VHX 6000). The dielectric constant of the glass fiber shells was approximated to be 4.5 from a study with similar fiber volume content and fiber orientation.^[^ ^52^ ^]^

The conductive layers of the FRP patches were composed of a 50 µm polyimide film (DuPont Kapton HN) with a dielectric constant of 3.4^[^ ^53^ ^]^ coated with the same silver particle solution as for the 3D printed prototypes.

The ground planes were made from 0.5 mm thick aluminum sheets for the helical antennas and from 0.5 mm thick Phosphor Bronze sheets for the patch antennas.

### Prototype Fabrication

The polymer substrates were 3D printed (Ultimaker 2+) in their flattened, unstretched (*α* = 0°) configuration from a CAD model generated using the Siemens NX software.^[^ ^54^ ^]^ A conductive trace was painted onto the TPU substrates to realize the antenna functional elements. For the helical antennas, the low bending stiffness of the 2D substrate allowed to deform it into a 3D cylindrical structure, with the seam joined via thermoplastic welding.

The FRP substrates were fabricated following a process modified from that developed by the authors in^[^ ^39^ ^]^ using a single autoclave cure of preimpregnated FRP laminates arranged into the metamaterial geometry. Casted silicone molds (SCS RTV 3428) were used to shape the composite laminates and Teflon release film (Airtech Release Ease 234) was used to separate neighboring hinges during cure. The assembly was constrained in a 3D‐printed PEEK outer frame and vacuum‐bagged for an autoclave cure of 2 h at 120 °C and a pressure of 4 bar. The thermal expansion of the silicone molds constrained by the PEEK frame provided sufficient pressure to ensure good consolidation of the composite material during cure.^[^ ^55^ ^]^ The polyimide film was bonded to the demolded FRP substrate post‐cure using an epoxy adhesive (EpoxAcast 690). The polyimide film was then cut to the desired geometry and coated with the conductive silver solution.

The exact geometry of the TPU prototypes was measured with a micrometer post‐fabrication to account for any manufacturing imperfections during the 3D‐printing process. The dielectric and conductor geometry of the FRP patch antennas were measured using optical microscopy (Keyence VHX 6000) to account for imperfections stemming from the silicone molding procedure. The average values from these measurements are those quoted in this work and used for the simulation of performance.

### Experimental Characterization

The antenna prototypes were experimentally characterized using a Vector Network Analyzer (VNA) (HP8753E 30 kHz to 6 GHz). The antennas were placed on top of a ground plane and fed with a 50 Ω coaxial cable attached to the VNA. Paperboard cylinders with varying diameters were inserted into the helical antennas to maintain a desired shape and the reflection coefficient magnitude was measured using the VNA.

The patch antennas were placed into 3D‐printed PLA stretching rigs to obtain the stretched configurations. The TPU substrate was stretched by pinning its four corner segments into predefined slots in the diagonal rails of the rig (Figure 3C). Due to the flexibility of the slender hinges in the FRP‐based substrates, a uniform displacement was required in the direction of stretching to result in a uniform expansion of the substrate rather than localized deformations. Therefore, 3D‐printed hooks were used to interface with segments along the positive and negative y‐boundaries of the FRP substrate (Figure S4, Supporting Information). The hooks were allowed to freely slide along a rail that was pinned at various y‐locations to apply uniaxial strain to the FRP substrate.

The radiation pattern, gain, and efficiency measurements of the patch antennas were completed at the antenna measurements chamber (anechoic chamber) of the American University of Beirut. The patch antennas were placed vertically on a foam column at a height of 136 cm from the ground, over a turning table that rotates 360°. The patch antennas were positioned facing a dual‐polarized horn antenna from ETS Lindgren (3164‐05 open boundary quad‐ridged horn) at a distance of 3.4 m. The antennas were connected through hidden RF cables to a Keysight PNA‐X 10 MHz to 67 GHz network analyzer^[^ ^56^ ^]^ on the exterior of the chamber and the computer controlling unit, which displays and saves the various plane cuts of the antenna radiation pattern.

For each reconfigurable state, the PNA‐X feeds the antenna with an RF signal at the corresponding frequency of operation with an input power of 0 dBm. The antenna under test radiates its corresponding signal that is received by the horn antenna facing it. The horn antenna routes the received signal into the controlling computer where the radiation pattern is adequately plotted.

### Simulation of Performance

All antenna performance in this work was simulated using ANSYS Electronics Desktop.^[^ ^46^ ^]^ The geometries of the antennas in the undeformed and deformed configurations were created using a parameterized model in the Siemens NX CAD software and imported into Electronics Desktop for analysis. The CAD model assumed purely kinematic deformations with a rotational mechanism at the center of each hinge. Electronics Desktop achieves a 3D simulation of the entire structure in its different configurations while relying on the finite element method to compute the various electromagnetic performance indicators such as reflection coefficient, as well as radiated electric and magnetic fields, among many others.

## Conflict of Interest

The authors declare no conflict of interest.

## Supporting information

Supporting Information

## Data Availability

The data that support the findings of this study are available from the corresponding author upon reasonable request.
