# Supplementary material for: Electromagnetic Reconfiguration Using Stretchable Mechanical Metamaterials
Source: Adv Sci (Weinh). 2023 Jan 4;10(6):2203376. doi: 10.1002/advs.202203376 (PMC9951316; doi:10.1002/advs.202203376)
Supplement: Supplementary file 1 — Supporting Information [file ADVS-10-2203376-s001.pdf]

ADVANCED SCIENCE
Open Access

Supporting Information

for *Adv. Sci.*, DOI 10.1002/advs.202203376

Electromagnetic Reconfiguration Using Stretchable Mechanical Metamaterials

Maria Sakovsky, Jan Negele and Joseph Costantine*

Supporting Information

Electromagnetic Reconfiguration Using Stretchable Mechanical Metamaterials

Maria Sakovsky[1], Jan Negele, Joseph Costantine*

M. Sakovsky, J. Negele
Department of Mechanical and Process Engineering, ETH Zurich, Leonhardstrasse 21, Zurich 8092, Switzerland
E-mail: msakovsky@ethz.ch

J. Costantine
Department of Electrical and Computer Engineering, American University of Beirut, Beirut 1107-2020, Lebanon

S1. Effect of metamaterial topology

We show that metamaterial antennas can be realized from arbitrary metamaterial patterns. Here, a reentrant honeycomb pattern is demonstrated for use in a stretchable helical antenna **(Figure S1A, B)**. The realized helix shows good matching to an input impedance of 50Ω over a large range of applied strains (0 < ε < 35%) and has an average frequency change metric of $\eta_{\varepsilon=20\%} = -0.59$ compared to $\eta_{\varepsilon=20\%} = -0.78$ for the standard helix reference (Figure S1C, D). The reentrant honeycomb geometry is selected such that its Poisson's ratio is roughly constant as a function of applied strain. The selected geometry results in a Poisson's ratio of $-0.25 < \nu < -0.3$ across the applied strain range.[1] The deviation from $\nu = -1$ results in a change of the helix aspect ratio, thereby changing the helix spacing relative to the diameter, as it is stretched. As a result, the average frequency change is lower than that observed for the rotating square pattern (Figure 2). Similar to the rotating square pattern, miniaturization

[1] Current affiliation: Department of Aeronautics and Astronautics, Stanford University, CA, USA

is observed compared to a standard helix due to the meandering of the surface current densities (Figure S1E).

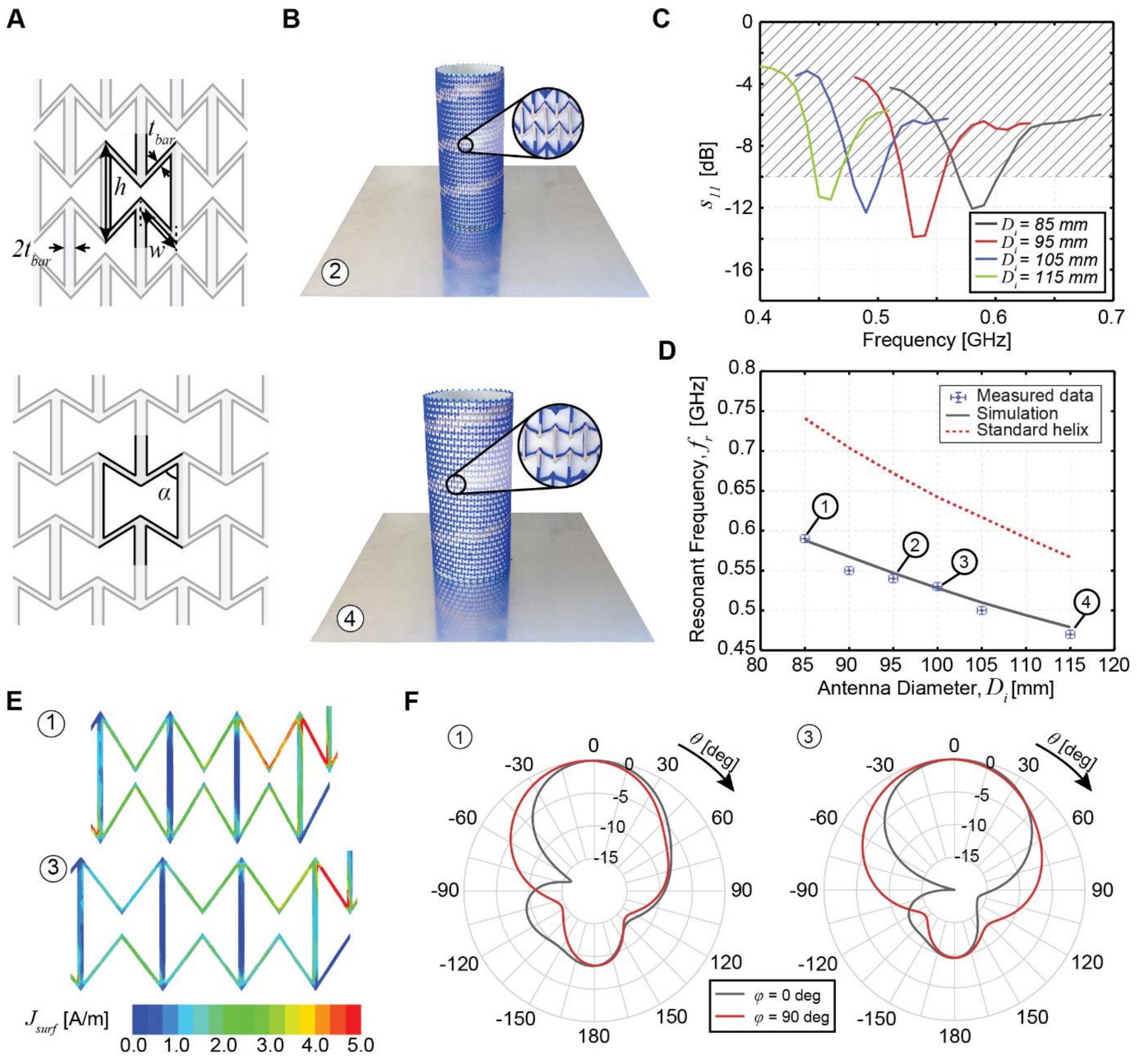

Figure S1. Frequency reconfigurable helical wire antenna using TPU re-entrant honeycomb metamaterial substrate. (A) Schematic of metamaterial geometry. The geometry is parametrized through the honeycomb height, h, the diagonal length, w, and the bar thickness, t_{bar}. The degree of stretching is characterized by the angle of rotation of the diagonals, α. **(B)** The prototype is pictured in two stretched configurations. The prototype geometry is as follows: h = 13.2 mm, w = 6.6 mm, t_{bar} = 0.3 mm, t_s = 1.9 mm, L_g = 400 mm, n_{turns} = 3.0, and $n_r \times n_z$ = 40×30. **(C)** Measured reflection coefficient magnitude for several patch configurations. **(D)** Demonstration of operating frequency reconfiguration upon stretching. The error bars represent the experimental uncertainty of the measurements. **(E)** Simulated surface current distribution with 0° phase in undeformed (1) and deformed (3) configurations. **(F)** Radiation pattern elevation cuts in undeformed (1) and deformed (3) configurations provided at the respective resonant frequency.

The helix radiates in the axial mode with an increase in 3 dB beamwidth from 88° to 94° upon stretching (Figure S1F). By comparison, the rotating square helix in Figure 2

maintains a constant 3 dB beamwidth of 75°. As a result, control of S/D_i as a function of ε can be used for radiation pattern reconfiguration from axial to broadside radiation.[2] We find a peak realized gain for the helix of 6.4 dB when unstretched and 5.8 dB when stretched to D_i = 100 mm. This is higher than the helix realized from the rotating square metamaterial, despite having a slightly lower number of turns (n_{turns} = 3.0 compared to n_{turns} = 3.2 for the helix in Figure 2).

Despite the lower frequency change, this substrate is advantageous due to higher achievable strains and control over biaxial deformation.

S2. Characterization of auxetic behavior of the FRP substrate

The mechanical performance of the FRP metamaterial substrate and antennas is characterized using quasi-static geometrically non-linear finite element simulations in Abaqus/Standard. The deformation and reaction forces upon uniaxial stretching are shown in **Figure S2** for the FRP substrate without the conductor as well as for the full antenna from Figure 4. It can be seen that the Poisson's ratio of the full antenna stays within 1% of the desired behavior of $\nu = -1$ up to strains of $\varepsilon = 40\%$. This is achieved by using a thinner hinge material relative to the segment material, thereby localizing the deformation to the hinges.[3]

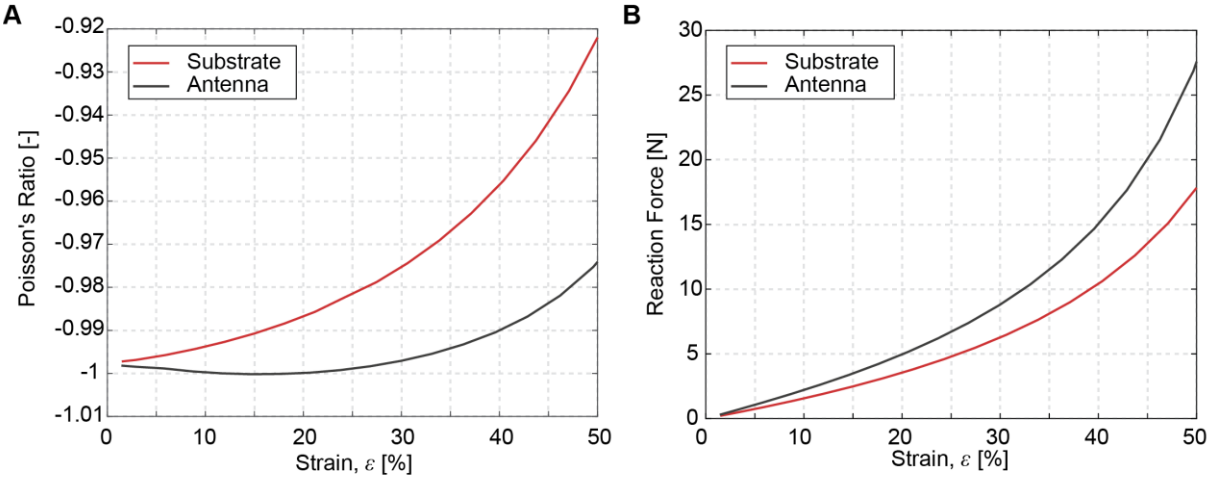

Figure S2. Simulated mechanical performance of FRP metamaterial antenna with $n = 5, \rho = 0.04$. (A) Poisson's ratio for substrate and antenna. **(B)** Forces required to apply uniaxial stretching of substrate and antenna.

S3. Homogenized dielectric properties of metamaterial substrates

To derive homogenized dielectric properties for the TPU and FRP substrates as they are stretched, we use the concept of capacitances in series and parallel. This analysis neglects any gaps in the conductor and focuses on the dielectric layer of the patch antenna. This layer is a combination of the substrate material in parallel with the air gaps introduced during stretching. In addition, the TPU substrate contains a small air gap between the ground plane and substrate (0.1 mm) due to the feed, while the FRP patches contain a 50 μm polyimide film onto which the conductor is coated. The simplified models are shown in **Figure S3**.

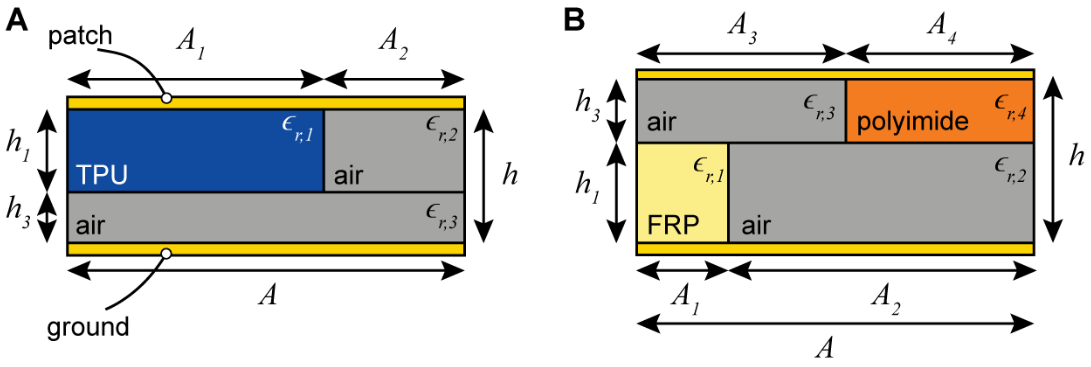

Figure S3. Schematic of simplified model used to compute effective dielectric properties of substrate. (A) TPU metamaterial. **(B)** FRP metamaterial.

We start with homogenization of the TPU dielectric (Figure S3A). Any dependence on the patch side length, L_p, is explicitly noted. The TPU and air gap layers are denoted by subscripts 1 and 2, respectively, and are treated as parallel capacitances,

$$
\begin{aligned}
C_{12} &= C_1 + C_2 \\
&= \frac{\epsilon_{r,1}\epsilon_0 A_1(L_p)}{h_1} + \frac{\epsilon_{r,2}\epsilon_0 A_2(L_p)}{h_1} \\
&= \frac{\epsilon_0 A(L_p)}{h_1}\left(\epsilon_{r,1}V_1(L_p) + \epsilon_{r,2}V_2(L_p)\right) \\
&= \frac{\epsilon_0 A(L_p)}{h_1}\epsilon_{r,12}(L_p)
\end{aligned}
$$

where $V_i = A_i/A$ is the fraction of area occupied by component i. This homogenized layer can be treated in series with the air gap below,

$$
\begin{aligned}
\frac{1}{C} &= \frac{1}{C_3} + \frac{1}{C_{12}} \\
&= \frac{h_3}{\epsilon_{r,3}\epsilon_0 A(L_p)} + \frac{h_{12}}{\epsilon_{r,12}(L_p)\epsilon_0 A(L_p)} \\
&= \frac{h}{\epsilon_0 A(L_p)}\left(\frac{d_3}{\epsilon_{r,3}} + \frac{d_1}{\epsilon_{r,12}(L_p)}\right) \\
&= \frac{h}{\epsilon_0 A(L_p)}\frac{1}{\epsilon_r(L_p)}
\end{aligned}
$$

where $d_i = h_i/h$ is the height fraction of layer i.

The initial gaps for the hinges of the TPU substrate and any deformation of the TPU during stretching are assumed to be negligible, employing a purely kinematic model for the analysis. Therefore, the area fractions of metamaterial TPU layer are given by,

$$V_1(L_p) = \left(\frac{L_p^0}{L_p}\right)^2$$

$$V_2(L_p) = 1 - V_1(L_p)$$

For the FRP metamaterial substrates (Figure S3B), the homogenization of the FPR layer follows exactly that of the TPU substrate,

$$C_{12} = \frac{\epsilon_0 A(L_p)}{h_1}\epsilon_{r,12}(L_p)$$

Similarly, the polyimide layer can be homogenized as capacitances in parallel,

$$C_{34} = \frac{\epsilon_0 A(L_p)}{h_3}\epsilon_{r,34}(L_p)$$

Lastly, the FRP metamaterial and polyimide layers are treated in series,

$$
\begin{aligned}
\frac{1}{C} &= \frac{1}{C_{12}} + \frac{1}{C_{34}} \\
&= \frac{h}{\epsilon_0 A(L_p)}\left(\frac{d_1}{\epsilon_{r,12}(L_p)} + \frac{d_3}{\epsilon_{r,34}(L_p)}\right) \\
&= \frac{h}{\epsilon_0 A(L_p)}\frac{1}{\epsilon_r(L_p)}
\end{aligned}
$$

The area fractions of the FRP metamaterial are estimated based on the prescribed geometry assuming a purely kinematic model for stretching,

$$V_1(L_p) = \left(\frac{n}{L_p}\right)^2 \left[2\pi r_s + 4\left(\frac{L_0}{n} - t_{hub} - 2r_s\right)\right] t_{hub} + 4(r_s + 0.5t_{hub})t_{hinge}$$

$$V_2(L_p) = 1 - V_1(L_p)$$

where, t_{hub} and t_{hinge} are the thicknesses of the hub and hinge regions of the metamaterial, respectively. The polyimide layer fully covers the surface at L_p^0 for the FRP prototype in Figure 4. Therefore,

$$V_4(L_p) = \left(\frac{L_p^0}{L_p}\right)^2$$

$$V_3(L_p) = 1 - V_4(L_p)$$

S4. Details of FRP metamaterial antennas and testing rig

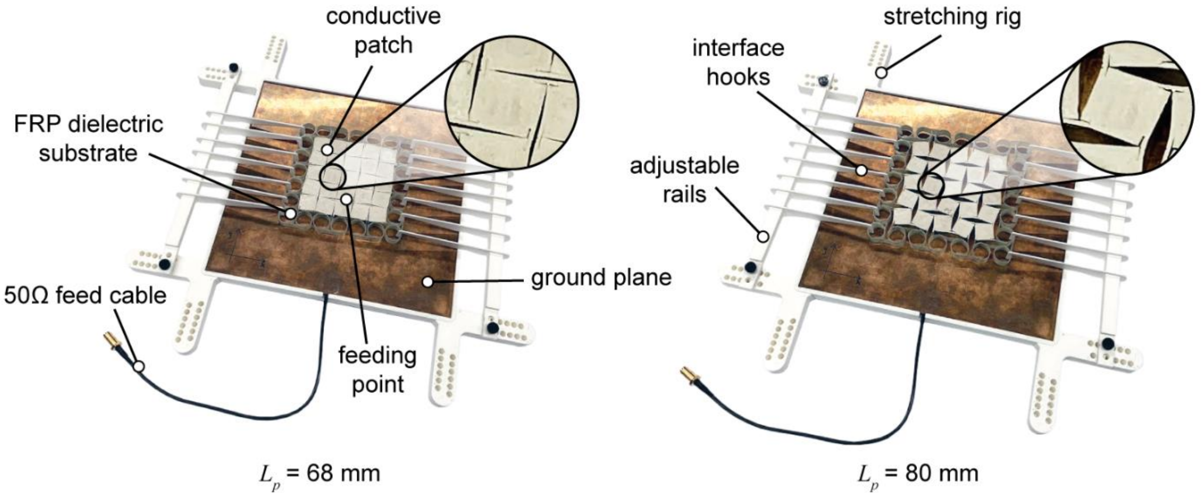

Figure S4. Zoomed in photographs of the FRP antenna in Figure 4 with its stretching rig.

S5. Experimental verification of simulated radiation patterns

Radiation patterns have been measured experimentally (Methods) for the FRP patch antennas to verify simulations **(Figure S5)**. Excellent agreement with simulations is observed with a slight increase in backlobe radiation seen in the measurements, which is expected due to constraints from the fabrication process and measurement limitations.

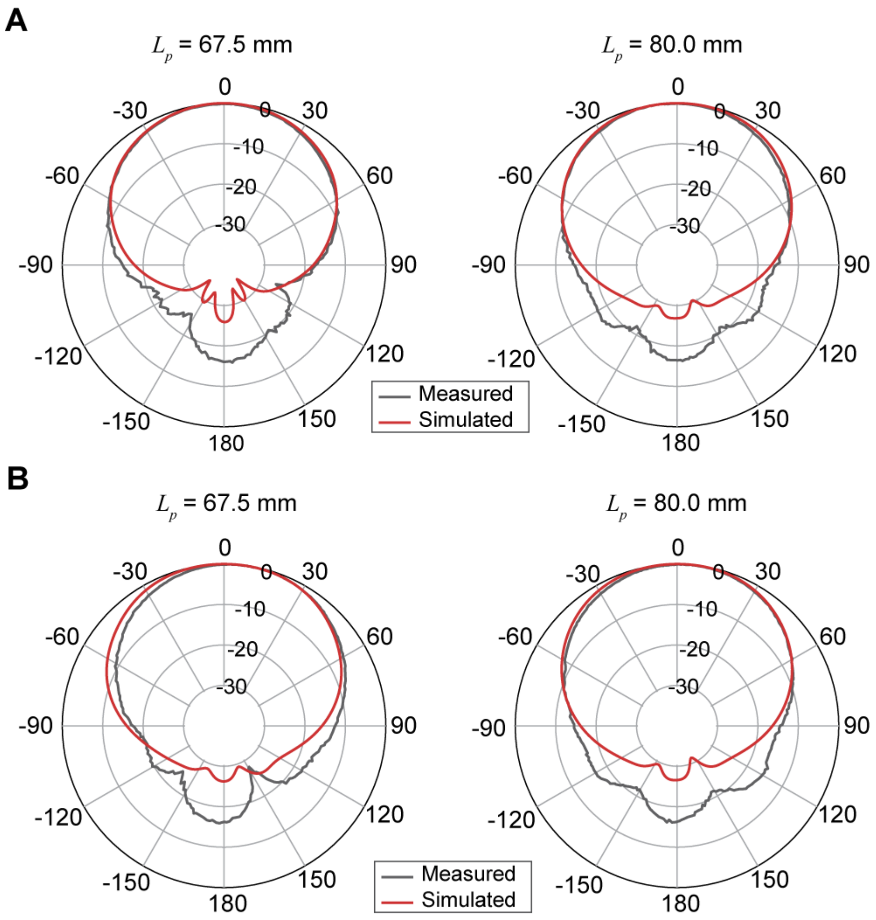

Figure S5. Experimental verification of simulated antenna radiation patterns. (A) FRP patch antenna with $n = 5, \rho = 0.04$ measured at 1.43GHz for L_p = 67.5 mm and 1.23 GHz for L_p = 80.0 mm. **(B)** FRP patch antenna with $n = 5, \rho = 0.3$ measured at 1.34 GHz for L_p = 67.5 mm and 1.21 GHz for L_p = 80.0 mm.

S6. Parameters affecting the frequency change metric of a patch antenna

The thickness and length of the hinges connecting metamaterial segments influence the impedance of the connections, which has an impact on the antenna's radiation characteristics. The effect of these parameters on the operating frequency of the antenna upon stretching are shown in **Figure S6**. The study is performed for an FRP metamaterial patch with L_p^0 = 67 mm, h = 8 mm, n = 5, ρ = 0.25. It is seen that $\eta_{\varepsilon=20\%} \propto l_h/t_h$, similarly to the impedance of the hinge.[4] The changes seen are relatively small compared to the parametric study presented in Figure 6. Furthermore, the distribution of surface currents shows only a small dependence on hinge parameters. Therefore, this effect is not investigated further. To ensure that this does not affect the study in Figure 6, l_h/t_h is kept constant for all values of n and ρ.

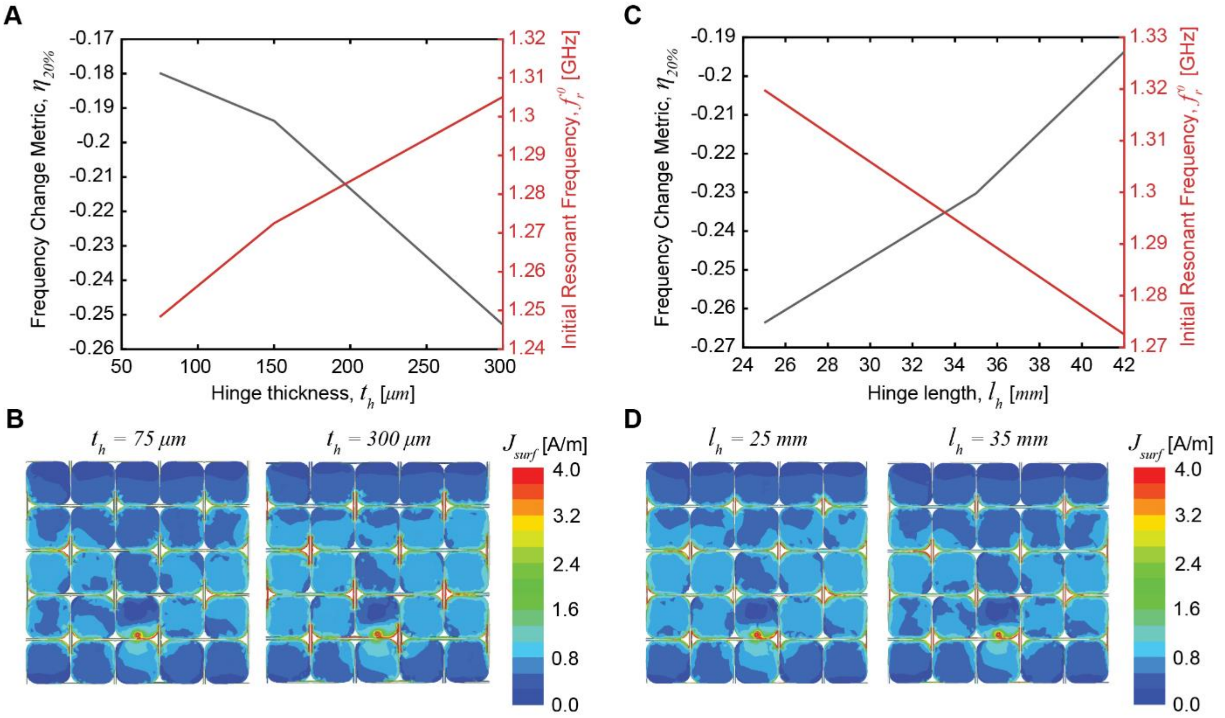

Figure S6. Metamaterial geometric parameters affecting the frequency change metric. (A) Influence of hinge thickness, t_h, on the frequency reconfiguration. **(B)** Simulated surface current densities with 0° phase for t_h = 75 µm and t_h = 300 µm. **(C)** Influence of hinge length, l_h, on the frequency reconfiguration. **(D)** Simulated surface current densities with 0° phase for l = 25 mm and l_h = 35 mm.

S7. Repeatability of frequency reconfiguration under cyclic loading

We demonstrate the repeatability of the frequency reconfiguration for the FRP mechanical metamaterial antennas. Specifically, we study the frequency reconfiguration for the prototype with n = 5, ρ = 0.3 (Figure 5A, gray line) under repeated mechanical loading (to ε = 15%) and unloading (to ε = 1.5%). Approximately 2 - 3 mins elapse between each stretching and unstretching operation and we conduct 10 repeated cycles on the first day of testing followed by 5 additional cycles after 72 hours.

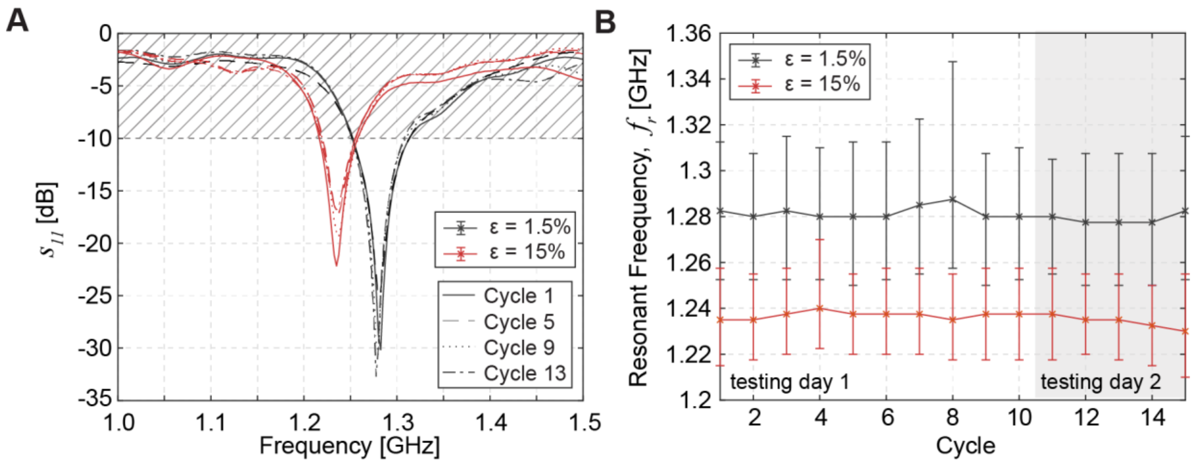

Figure S7. Repeatability of frequency reconfiguration for patch antenna with FRP substrate and $\boldsymbol{n}$ = 5, $\boldsymbol{\rho}$ = 0.3. (A) Reflection coefficient amplitudes for select cycles for applied strains of ε = 1.5% and ε = 15%. **(B)** Resonant frequency as a function of the number of reconfiguration cycles. The bars denote the operating bandwidth of the patch antenna.

Figure S7A shows the measured reflection coefficient magnitudes for select cycles in the stretched and unstretched configurations. Excellent repeatability of the frequency change is observed. This is more clearly illustrated in Figure S7B, which shows the resonance frequency in the two configurations as a function of the number of stretching cycles. The bars denote the operating bandwidth of the patch antenna in each configuration.

For a given antenna configuration, the resonant frequency fluctuates by less than 1% between the maximum and minimum measured values across all cycles. This is a small fraction of the bandwidth of the antenna for the corresponding configuration. We also observe that any fluctuations across cycles are random, with no consistent upward or downward trend. Additionally, there is no loss of matching of the antenna in either configuration due to cycling.

The stretching history (i.e. acyclic order of reconfiguration) can impact the observed frequency adaptation of the antenna with fluctuations of the resonant frequency by up to ±2.5% compared to the average values in Figure S7B. While these fluctuations are larger than those for cyclical loading, they are within the bandwidth of the antenna for the given configuration and hence are deemed acceptable. The cause of these fluctuations is viscoelastic effects of the FRP composite material.[5] We minimize these effects by using short cycle times and prescribed displacement boundary conditions rather than prescribed loading. In addition, repetition of the tests after a long relaxation period of 72 hours does not yield any significant shift in the operating frequency.

References

[1] H. Wan, H. Ohtaki, S. Kotosaka, G. Hu, *Eur. J. Mech. A/Solids* **2004**, *23*, 95.
[2] W. Lin, H. Wong, in *2015 9th Eur. Conf. Antennas Propagation, EuCAP 2015*, EurAAP, **2015**.
[3] M. Sakovsky, P. Ermanni, *Compos. Struct.* **2020**, *246*, 112390.
[4] C. A. Balanis, *Antenna Theory: Analysis and Design*, John Wiley And Sons, Hoboken, New Jersey, **2005**.
[5] K. Kwok, S. Pellegrino, *52nd Struct. Struct. Dyn. Mater. Conf.* **2011**, AIAA 2011.
